# Neoadjuvant chemotherapy followed by curative-intent surgery for perihilar cholangiocarcinoma based on its anatomical resectability classification and lymph node status

**DOI:** 10.1186/s12885-020-06895-1

**Published:** 2020-05-11

**Authors:** Naohisa Kuriyama, Masanobu Usui, Kazuaki Gyoten, Aoi Hayasaki, Takehiro Fujii, Yusuke Iizawa, Hiroyuki Kato, Yasuhiro Murata, Akihiro Tanemura, Masashi Kishiwada, Hiroyuki Sakurai, Shugo Mizuno, Shuji Isaji

**Affiliations:** Department of Hepatobiliary Pancreatic and Transplant Surgery, Mie University Graduate School of Medicine, 2-174 Edobashi, Tsu city, Mie 514-8507 Japan

**Keywords:** Perihilar cholangiocarcinoma, Resectability classification, Neoadjuvant chemotherapy

## Abstract

**Background:**

The prognosis of patients with perihilar cholangiocarcinoma have been unsatisfactory. We established new anatomical resectability classification for patients with localized perihilar cholangiocarcinoma and performed neoadjuvant chemotherapy followed by curative-intent surgery based on its resectability classification and lymph node status to improve prognosis. This study aimed to clarify the long-term outcomes and validation of our strategy.

**Methods:**

Between September 2010 and August 2018, 72 consecutive patients with perihilar cholangiocarcinoma were classified into three groups: Resectable (R = 29), Borderline resectable (BR = 23), and Locally advanced (LA = 20), based on the two factors of tumor vascular and biliary extension. R with clinically lymph node metastasis, BR, and LA patients received neoadjuvant chemotherapy using gemcitabine plus S-1.

**Results:**

Forty-seven patients (65.3%) received neoadjuvant chemotherapy: R in 8, BR in 21, and 18 in LA, respectively. Fifty-nine patients (68.1%) underwent curative-intent surgery: R in 26, BR in 17, and LA in 6. Five-year disease-specific survival was 31.5% (median survival time: 33.0 months): 50.3% (not reached) in R, 30.0% (31.4 months) in BR, and 16.5% (22.5 months) in LA, which were relatively stratified. Among 49 patients with resection, disease-specific survival was 43.8% (57.0 months): 57.6% (not reached) in R, 41.0% (52.4 months) in BR, and 0% (49.4 months) in LA, which were significantly good prognosis compared to 23 patients without resection (17.2 months). Multivariate analysis identified preoperative high carcinoembryonic antigen levels (more than 8.5 ng/ml) and pT4 as independent poor prognostic factor of patients with resection.

**Conclusion:**

Neoadjuvant chemotherapy based on resectability classification and lymph node status was feasible, and was considered efficacious in selected patients.

## Background

In the localized perihilar cholangiocarcinoma, negative tumor margin resection contributes to get an opportunity for long recurrence free survival. In the past two decades, advances in diagnostic and surgical techniques have improved surgical outcomes and survival rates [1]. However, the prognosis of the resected perihilar cholangiocarcinoma patients with lymph node (LN) metastasis has not been improved. Therefore, we should not only perform negative tumor margin resection, but also establish effective adjuvant and/or neoadjuvant therapy for the localized perihilar cholangiocarcinoma with LN metastasis.

The role of adjuvant chemotherapy (AC) for resected bile duct cancer (BTC) is controversial. Although 3 phase-III randomized trials have been explored in the adjuvant setting for BTC [2–4], the positive effects of AC were not well defined.

In terms of neoadjuvant chemotherapy (NAC) for localized BTC, there are a few retrospective small reports [5–7]. They considered that NAC followed by curative-intent surgery might offer downstaging for initially resectable BTC and conversion surgery for initially unresectable BTC, resulted in improving prognosis. Recently, using the large National Cancer Database data, a propensity score matched analysis using resected patients with cholangiocarcinoma indicated that patients who received NAC alone had a superior overall survival compared to those who received AC alone [8]. This study implied the benefit of NAC for selected patients with cholangiocarcinoma as well as other malignancies including pancreatic and breast cancer.

In the field of pancreatic ductal adenocarcinoma (PDAC), localized tumors are anatomically classified as resectable (R), borderline resectable (BR), or locally advanced (LA) based on the likelihood of a positive margin resection. Neoadjuvant chemo and/or radiotherapy is introduced PDAC patients based on its classification. In our institution, neoadjuvant gemcitabine based chemoradiotherapy for advanced PDAC based on its resectability has been introduced since 2005 and its prognosis has been improved [9]. Therefore, we originally established the anatomical resectability classification for localized perihilar cholangiocarcinoma according to surgical points of view from biliary and vascular extension as well as PDAC. As extrahepatic bile duct cancer partly shares embryological, clinical and pathological features with PDAC [10], this favorable effect of gemcitabine prompted to conduct NAC using gemcitabine plus S-1 in patients with perihilar cholangiocarcinoma.

This study aimed to evaluate the feasibility of anatomical resectability classification and the efficacy of NAC followed by curative-intent surgery based on its classification and LN status.

## Methods

Between September 2010 and August 2018, 72 consecutive patients with localized perihilar cholangiocarcinoma, who were all eligible patients identified by us, had been enrolled for our institutional treatment protocol based on our established resectability classification from surgical points of view. The diagnosis of perihilar cholangiocarcinoma was confirmed by means of cytological analysis of bile juice or histological analysis of biopsy specimens obtained using endoscopic retrograde cholangiography (ERC). Patients were excluded when they showed evident distant metastatic lesions at the time of enrollment. On the basis of our resectability classification mentioned below, the 72 patients were classified into the three groups: R (*n* = 29), BR (*n* = 23), and LA (*n* = 20). Of them, 43 were men and 29 were women, with an average age of 71 years (range 44–87 years).

The clinical and follow-up information was extracted from a prospectively maintained database at the department of hepatobiliary pancreatic and transplant surgery, Mie university hospital, and verified by reviewing patient medical records. The day of final follow-up was March 31, 2019.

### Resectability classification of localized perihilar cholangiocarcinoma from surgical points of view

In 2010, we established our own anatomical resectability classification for localized perihilar cholangiocarcinoma which consists of the three categories: R, BR, and LA according to surgical points of view from biliary and vascular factors **(**Table 1**)**. Initial resectability classification was performed based on initial dynamic multidetector-row computed tomography (MDCT) findings before biliary drainage at the visit to our hospital. High resolution CT allows us to make accurate depiction of a thickened bile duct wall and tumor spread into liver parenchyma or hilar vessels [11]. This was followed by ERC and intraductal ultrasonography (IDUS) to evaluate tumor biliary extension. Selective cannulation under ERC was performed to ascertain segmental duct evaluation. After a diagnostic ERC and IDUS, biopsies of a root of posterior bile duct, a root of B4, and bifurcation of B2 and B3 were perform to obtain histological evidence of biliary extension for surgical planning. Finally, endoscopic retrograde biliary drainage tubes (plastic stents) were inserted into the future remnant liver in the patients with obstructive jaundice. In terms of vascular factor of portal vein (PV) and hepatic artery (HA), contact with the tumor greater than 180 degree, irregular encasement or occlusion were all considered as corresponding to vascular invasion [12]. Additionally, positron emission tomography computed tomography (PET-CT) and MDCT were used for the evaluation for LN metastasis and distant metastasis.

**Table 1:** Resectability classification of localized perihilar cholangiocarcinoma from surgical points of view

Resectability	Biliary factor		Vascular factor
**Resectable (R)**	Curative resection **can be** obtained by either side of right or left TSN or less hepatectomy	and	Combined VR with reconstruction of PV and/or HA is **not required** regardless of vascular invasion
**Borderline** **Resectable (BR)**	Curative resection **can be** obtained by either side of right or left TSN or less hepatectomy	and	Combined VR with safe reconstruction of PV and/or HA **can be** performed
**Locally advanced (LA)**	Curative resection **cannot be** obtained even by either side of right or left TSN	and/or	Combined VR with safe reconstruction of PV and/or HA **cannot be** performed

*TSN* trisectionectomy, *VR* vascular resection, *PV* portal vein, *HA* hepatic artery

Biliary factor is defined whether or not curative resection could be obtained by either side of right or left trisectionectomy or less hepatectomy. Vascular factor is defined whether or not combined vascular resection and reconstruction of PV and/or HA is required. Finally, the three classifications are determined by combination of biliary and vascular factors as follows.

R: curative resection can be obtained by either side of right or left trisectionectomy or less hepatectomy (biliary factor), and combined resection with reconstruction of PV and/or HA is not required regardless of vascular invasion (vascular factor).

BR: curative resection can be obtained by either side of right or left trisectionectomy or less hepatectomy (biliary factor), and combined vascular resection with safe reconstruction of PV and/or HA can be performed (vascular factor).

LA: curative resection cannot be obtained even by either side of right or left trisectionectomy (biliary factor), and/or combined vascular resection with safe reconstruction of PV and/or HA cannot be performed (vascular factor).

### Our institutional treatment protocol for patients with localized perihilar cholangiocarcinoma according to our resectability classification and LN status

**Fig. 1:**
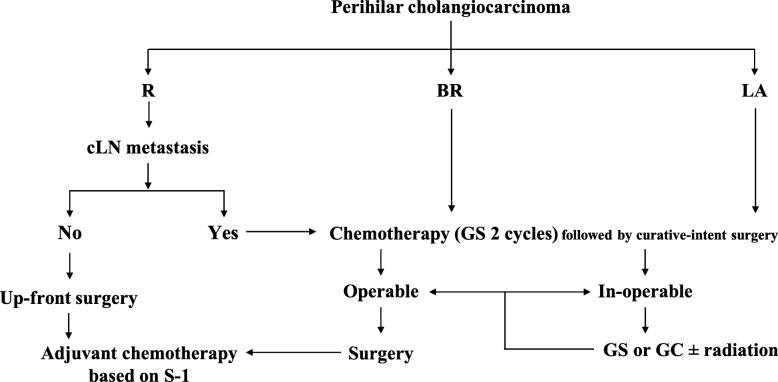
Our institutional treatment protocol for patients with localized perihilar cholangiocarcinoma according to our resectability classification. *R*: resectable, *BR*: borderline resectable, *LA*: locally advanced, *LN*: lymph node, *GS*: gemcitabine + S-1, *GC*: gemcitabine + cisplatin

**Fig. 2:**
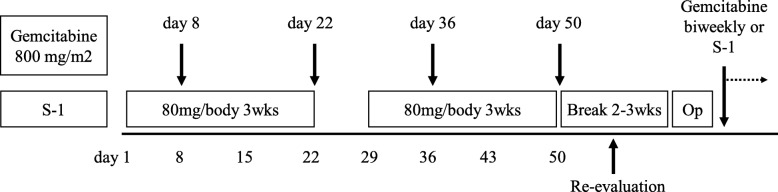
Protocol of 2 cycles Gemcitabine plus S-1 therapy

As shown in Fig. 1, up-front surgery was selected for the R patients without clinical evidence of LN metastasis based on the findings of PET-CT and MDCT. NAC was performed for the R with clinical evidence of LN metastasis, BR and LA patients. NAC regimen **(**Fig. 2**)** included 2 cycles of chemotherapy with gemcitabine (800 mg/m2 on days 7 and 21) plus S-1 (80 mg/body daily on days 1–21 every 4 weeks, GC) [13, 14]. After re-evaluation, the patients received curative-intent surgery when the tumor was determined resectable. When curative-intent surgery was determined impossible, GS therapy was continued, or chemotherapy protocol was changed to gemcitabine plus cisplatin (GC), with or without adding radiotherapy.

### Evaluation of tumor and host related factors, and toxicity grading of chemotherapy

In terms of tumor related factors, serum tumor markers, such as carcinoembryonic antigen (CEA) and carbohydrate antigen 19–9 (CA19–9), were measured before the initial treatment and curative-intent surgery. In terms of host related factors, inflammatory/immunonutritional factors, such as neutrophil-to-lymphocyte ratio (NLR), and prognostic nuritional index (PNI), were evaluated before the initial treatment and curative-intent surgery. Inflammatory/immunonutritional factors have been reported to predict the prognosis of patients with various malignancies [15].

Toxicity of chemotherapy was categorised according to the National Cancer Institute’s Common Toxicity Criteria for Adverse Events, version 3.0. Toxicity was recorded continuously during treatment and serious adverse events were monitored throughout.

### Indication of curative-intent surgery, surgical procedure, and postoperative complications

Based on preoperative imaging studies, we determined which side of hepatectomy with caudate lobectomy could be performed to obtain curative resection according to the biliary and vascular factors. In terms of biliary extension, right hepatectomy was applied to Bismuth type I, II, and IIIa tumors. Left hepatectomy was applied to Bismuth type IIIb tumors. When the tumor obviously extended over the second order biliary radicles, such as Bismuth type IV tumors, trisectionectomy or central bisectionectomy was selected. However, patients with obvious invasion of the right side of the umbilical portion (U portion) and the left side of the origin of the right posterior portal vein (P portion) were contraindication for surgery. In terms of vascular invasion, it was critical point to secure at least 5 mm tumor free hepatic margin of PV and/or HA in the remnant liver side for safe vascular resection and reconstruction. Vascular extension beyond the second branch of PV and/or HA was contraindication because safe vascular resection and reconstruction were usually impossible. When patients had sufficient remnant liver function for proposed operation which achieve R0 resection even if we found tumor progression, curative-intent surgery was underwent because R0 resection contributed to get an opportunity for long recurrence free survival. However, it was difficult to evaluate biliary and vascular extension after biliary drainage. Occasionally, it was up to intraoperative judgment in order not to miss the opportunity of R0 resection.

For almost LA patients, they remained unresectable at the time of reevaluation after 2 cycles of chemotherapy, and thus we continued chemotherapy using GS or GC regimen with or without adding external radiotherapy, followed by every three-month interval of reevaluation to seek the timing of curative-intent surgery. When the biliary and vascular factors of unresctability were determined to be overcome, we decided to perform curative-intent surgery.

Combined with the above mentioned biliary and vascular factors for hepatectomy, the type of hepatectomy was finally determined by the remnant liver function. The future remnant liver function was determined by multiplying the future functional remnant liver ratio (f-rem) by the indocyanine green plasma clearance rate (KICG) (f-rem-KICG). The f-rem was calculated by the fusion image of MDCT and hepatic uptake ratio of 99mTc-GSA scintigraphy at 15 min using 3D simulation software (Synapse Vincent; Fujifilm, Tokyo, Japan) [16]. Patients with the f-rem-KICG of less than 0.05 was not indication for major hepatectomy based on the previous paper [17]. Portal vein embolization was indicated when the future remnant liver volume was estimated as less than 40%. Occasionally, limited resection was selected for patients with insufficient liver function for major hepatectomy and poor performance status [18].

Postoperative complications including morbidity and mortality were graded according to the Clavien-Dindo classification [19].

### Pathological assessment

The resected specimens were fixed in a formalin solution, sectioned approximately 5-mm intervals and embedded in paraffin blocks. A 3-μm section was obtained from each block and stained with hematoxylin and eosin. All specimens underwent routine histopathological work-up according to the American Joint Committee on Cancer staging system, 7th edition. Pathological differentiation, degree of LN metastasis, and assessment of residual tumor and so on was evaluated by an experienced pathologist. R1 status was defined based on microscopic tumor exposure at any biliary, vascular, and hepatic paremchema resection margin of the surgical specimen. R2 status was also defined based on macroscopic tumor exposure or distant metastasis including intrahepatic metastasis.

### Postoperative chemotherapy and follow-up

From 4 to 6 weeks after resection, we started the AC and continue at least 6 months. Chemotherapy regimen was gemcitabine at a dose of 800 mg/m^2^ biweekly, from February 2005 to May 2013, and S1 orally twice daily at a dose of 60 mg/m^2^/day on days 1 through 28 of a 42-day cycle from June 2013 to March 2019. Depending on patient tolerability of AC regimen, we changed the regimen from gemcitabine to S1 or vice versa. After operation, all patients were evaluated as follows: physical examination every month; laboratory tests including 12 serum levels and tumor marker levels (CEA and CA19–9) every 2 or 3 months; and 4-phasic contrast-enhanced MDCT every 4 months within 2 years and thereafter every 6 months. If the serum levels of the tumor markers increased, the patients were immediately evaluated by MDCT.

### Statistical analysis

Continuous and categorical variables were expressed as median (range) and were compared using the Mann-Whitney test and chi-square test. In all patients who came for re-assessment, the date of the initial treatment was chosen as the starting point for the measurement of survival time. Patients who were alive or had died of a cause other than perihilar cholangiocarcinoma were censored for analysis of disease-specific survival (DSS) and median survival time (MST: months). Survival was calculated using the Kaplan-Meier method and was compared between the groups using the log-rank test. The day of final follow-up was March 31, 2019. All variables were dichotomized for analyses. A multivariate analysis was performed using Cox proportional hazard model. Variables with a significance of *p* < 0.05 in the univariate analysis were entered into the multivariate analysis. Comparisons were performed using the X2 test with Yates correction in the univariate analysis. All statistical analyses were performed using the SPSS version 24 (SPSS Inc., Chicago, III) software. A *p* value less than 0.05 was considered statistically significant.

## Results

### Flow diagram of the patients with localized perihilar cholangiocarcinoma

**Fig. 3:**
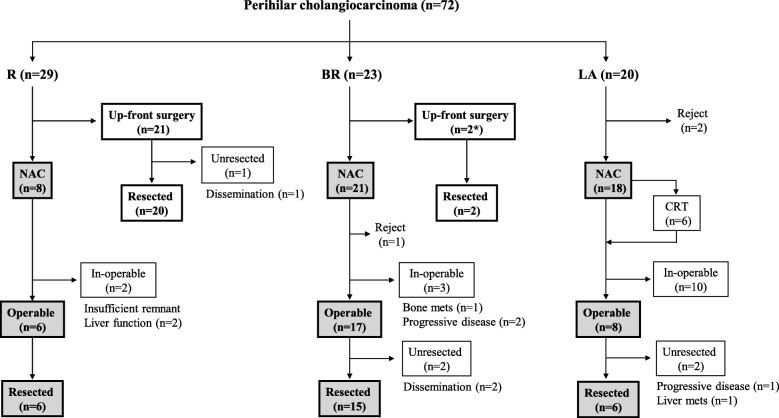
Treatment flow diagram for the patients with localized perihilar cholangiocarcinoma according to the resectability classification. *R*: resectable, *BR*: borderline resectable, *LA*: locally advanced, *NAC*: neoadjuvant chemotherapy followed by curative-intent operation, *CRT*: chemoradiation therapy

The enrolled 72 patients with localized perihilar cholangiocarcinoma had been classified into the three groups: R (*n* = 29), BR (*n* = 23), and LA (*n* = 20). Figure 3 shows the flow diagram of treatment for these patients according to the resectability classification. Among 29 R patients, up-front surgery was performed in 21, of whom 20 could undergo resection, and NAC was selected in 8 with suspected regional LN metastasis, of whom 6 could undergo surgery. Among 23 BR patients, up-front surgery was performed in 2 who had repeated cholangitis in one and had biliary duct injury during preoperative ERC in one, respectively, both of whom could undergo surgery, and NAC was selected in 21, of whom 15 could undergo surgery. Among 20 LA patients, excluding 2 with rejection, NAC was performed in 18, of whom 6 could undergo surgery.

### Characteristics of the enrolled patients

Characteristics in three groups of R, BR and LA are summarized in Table 2. There were no differences between the three groups in age, gender, biliary drainage rate, initial inflammatory/immunonutritional factors (NLR and PNI), and levels of tumor markers (CEA and CA19–9). In terms of initial clinical staging, the rate of patients who were clinically diagnosed as cT4 was significantly higher in BR (16/23, 69.6%) and LA (17/20, 85.0%) groups than in R (2/29, 6.6%) group (*p* < 0.001). However, there were no differences between the three groups in clinical LN metastasis based on initial MDCT and PET-CT. The induction ratio of NAC was significantly lower in R (8/29, 27.6%) group than in BR (21/23, 91.3%) and LA (18/20, 90.0%) groups (*p* < 0.001). there were no differences among the three groups in completion rate of initial GS therapy and adverse reaction rate. The resection ratio was significantly higher in R (26/29, 89.7%) and BR (17/23, 73.9%) groups than in LA (6/20, 30.0%) group (*p* < 0.001).

**Table 2:** Characteristics of 72 patients according to the respectability classification

Factors	R (n = 29)	BR (n = 23)	LA (n = 20)	***p*** value
Age (years)	72 (44–87)	70 (51–80)	70 (45–85)	0.725
Gender (male / female)	15 / 14	12 / 11	16 / 4	0.094
Patients with biliary drainage (%)	25 (86.2%)	16 (69.6%)	17 (85.0%)	0.270
**Initial blood examination**				
Neutrophil-to-lymphocyte ratio (NLR)	2.03 (0.97–8.15)	2.21 (1.14–7.90)	3.15 (0.98–10.59)	0.083
Prognostic nuritional index (PNI)	42.5 (33.9–55.6)	44.6 (35.2–56.2)	42.4 (32.7–54.8)	0.566
CEA (mg/dl)	3.6 (0.7–38.4)	3.6 (0.9–16.3)	4.1 (1.6–65.7)	0.625
CA19–9 (U/ml)	100.7 (7.0–7898)	98.4 (1.0–9066)	208 (5.2–5265)	0.889
**Initial clinical staging**				
Patients with cT4 (%)	**2 (6.9%)**	**16 (69.6%)**	**17 (85.0%)**	< 0.001*
Patients with clinical LN metastasis (%)	7 (24.1%)	7 (30.4%)	10 (50.0%)	0.158
Patients with neoadjuvant GS therapy (%)	**8 (27.6%)**	**21 (91.3%)**	**18 (90.0%)**	< 0.001*
Completion rate of initial 2 cycles (%)	7/8 (87.5%)	20/21 (95.2%)	16/18 (88.9%)	
Median relative dose intensity of S-1 (%)	100%	100%	100%	
Median relative dose intensity of G (%)	101%	87.4%	81.8%	
Patients with adverse events (%)	7 (87.5%)	14 (66.7%)	14 (77.8%)	
Toxicity grade 1/2	4	2	6	
Toxicity grade 3/4 (grade 3 cholangitis)	3 (2)	12 (10)	8 (6)	
Patients with resection (%)	**26 (89.7%)**	**17 (73.9%)**	**6 (30.0%)**	< 0.001**

*R* resectable, *BR* borderline resectable, *LA* locally advanced * R vs. BR, UR, **R, BR vs. UR

*CEA* carcinoembryonic antigen, *CA19–9* carbohydrate antigen 19–9, *GS* gemcitabine plus S-1, *LN* lymph node, *G* gemcitabine

The total of 49 patients could undergo curative-intent surgery. Characteristics in three resected patient groups of R, BR and LA are summarized in Tables 3 and 4. The rate of patients who received NAC or chemoradiotherapy was significantly higher in BR (15/17, 88.2%) and LA (6/6, 100%) groups than in R (6/26, 23.1%) group (*p* < 0.001). In terms of pathological findings, the rate of patients who diagnosed as pT4 was significantly higher in BR (7/17, 41.2%) and LA (3/6, 50.0%) groups than in R (2/26, 7.7%) group (p < 0.001). However, there were no differences among the 3 groups in histological differentiation, LN metastasis, intrahepatic metastasis, and R0 resection rate. However, all four patients with limited resection such as hilar bile duct resection with or without S1 hepatectomy who were classified as Bismuth type I or II were belonged to the R group. Among them, 2 patients could not achieve R0 resection. Additionally, all four patients with pancreatoduodenectomy and hilar bile duct resection who were classified as Bismuth type I were also belonged to the R group. Unfortunately, 3 patients could not achieve R0 resection. Therefore, among 8 patients classified as R group who underwent curative-intent surgery without major hepatectomy, only 3 (37.5%) patients achieve R0 resection resulted in relative low R0 resection rate in the R group. In contrast, among 18 patients classified as R group who underwent curative-intent surgery using major hepatectomy, 13 (72.2%) patients could achieve R0 resection. In terms of intraoperative outcomes, there were no differences among the three groups in operation time and blood loss. The rate of patients who underwent combined PV resection and reconstruction was significantly higher in BR (12/17, 70.6%) and LA (3/6, 50%) groups than in R (4/26, 15.4%) group (*p* < 0.001). In terms of postoperative course, there were no differences among the three groups in postoperative complication, 90-day mortality, and induction rate of AC.

**Table 3:** Characteristics of 49 patients with resection

Factors	R (***n*** = 26)	BR (***n*** = 17)	LA (***n*** = 6)	***p*** value
Age (years)	72 (44–87)	70 (51–80)	65 (53–84)	0.478
Gender (male / female)	14 / 12	9 / 8	4 / 2	0.830
Biliary drainage (%)	24 (92.3%)	11 (64.7%)	5 (83.3%)	0.073
Adjuvant therapy (%)	**6 (23.1%)**	**15 (88.2%)**	**6 (100%)**	< 0.001*
Chemotherapy	6	15	4	
Chemoradiotherapy	0	0	2	
Portal vein embolization (%)	2 (7.7%)	4 (23.5%)	1 (14.3%)	0.344
**Preoperative blood examination**				
Neutrophil-to-lymphocyte ratio	2.31 (0.97–8.15)	2.43 (1.52–6.15)	3.86 (0.99–6.46)	0.276
Prognostic nuritional index	42.4 (33.9–55.6)	43.8 (36.3–50.1)	42.6 (32.7–46.8)	0.707
CEA (mg/dl)	3.0 (0.5–30.8)	3.7 (1.5–24.4)	2.6 (2.2–5.4)	0.644
CA19–9 (U/ml)	59.6 (12.6–11,659)	69.7 (1–1158)	196.6 (8.0–669.3)	0.715
**Histological differentiation**				0.473
G1/2 (%)	24 (96.0%)	15 (88.2%)	6 (100%)	
G3 (%)	1 (4.0%)	2 (11.8%)	0	
**Pathological finding**				
pT4 (%)	**2 (7.7%)**	**7 (41.2%)**	**3 (50.0%)**	0.013*
Lymph node metastasis (%)	9 (34.6%)	11 (64.7%)	3 (50.0%)	0.152
Intrahepatic metastasis (%)	1 (3.8%)	2 (11.8%)	0	0.457
**Residual tumor**				
R0 (%)	16 (61.5%)	12 (70.6%)	4 (66.7%)	0.828
R1	6	3	1	
R2	4	2	1	

*R*: resectable, *BR*: borderline resectable, *LA*: locally advanced * R vs. BR, UR

*CEA* carcinoembryonic antigen, *CA19–9* carbohydrate antigen 19–9, *R0* complete resection, *R1*: microscopic residual tumor resection, *R2*: macroscopic residual tumor resection or distant metastasis (intrahepatic metastasis)

**Table 4:** Characteristics of 49 patients with resection

Factors	R (n = 26)	BR (n = 17)	LA (n = 6)	*p* value
Operation time (min)	629 (427–943)	600 (383–728)	597 (395–663)	0.471
Blood loss (ml)	1385 (166–5166)	1720 (166–6059)	2383 (1443-4090)	0.485
**Type of hepatectomy**∗				
S1,2,3,4	8	9	2	
S1,2,3,4,5,8	1	2		
S1,5,6,7,8	5	5	3	
S1,4,5,6,7,8			1	
S1,4,5,8	1			
S1,5,8	1			
S1with hilar bile duct resection	2			
Hilar bile duct resection	2			
Pancreatoduodenectomy	4			
Hepatopancreatoduodnectomy	2	1		
Combined PV resection and reconstruction (%)	**4 (15.4%)**	**12 (70.6%)**	**3 (50.0%)**	**0.001**
Combined HA resection and reconstruction (%)	0	3 (17.6%)	1 (16.7%)	0.085
**Morbidity and Mortality**				
Complication (Clavien-Dindo≧III) (%)	14 (53.8%)	8 (47.1%)	3 (50.0%)	0.908
90-day mortality (%)	0	0	0	
**Adjuvant chemotherapy (%)**	14 (53.8%)	15 (88.2%)	4 (66.7%)	0.063

*R* resectable, *BR* borderline resectable, *LA* locally advanced * R vs. BR, UR

∗Expressed as Couinaud’s hepatic segments resected, PV: portal vein, HA: hepatic artery

### Survival analysis according to the resectability classification

**Fig. 4:**
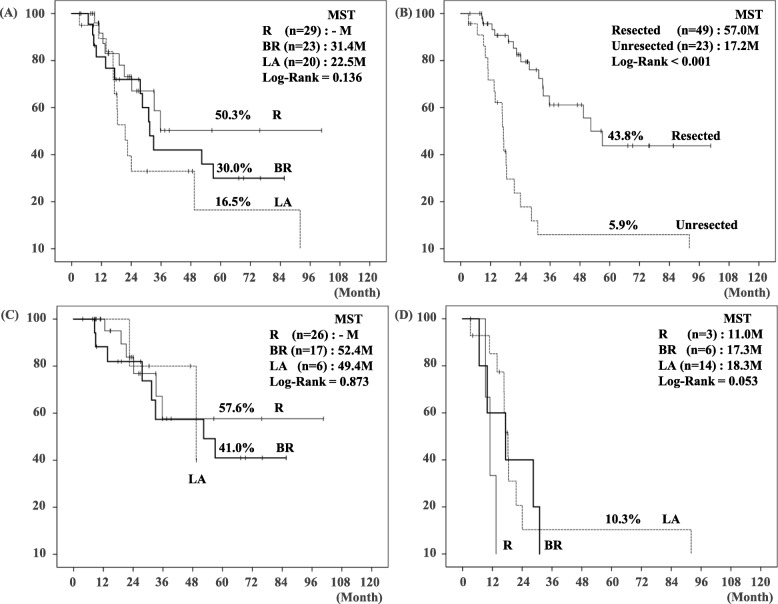
Disease-specific survival (DSS) curves in the 72 patients with localized perihilar cholangiocarcinoma. (**a**) Comparison with DSS among R, BR, and LA: There was no statistical difference of 5-year DSS (50.3% vs. 30.0% vs. 16.5%, *p* = 0.136, respectively). (**b**) Comparison with DSS between resected (*n* = 49) and unresected (*n* = 23) patients: there was significant difference of 5-year DSS (43.8% vs. 5.9%, *p* < 0.001). (**c**) Comparison with DSS among R, BR, and LA in patients with resection: There was no statistical difference of 5-year DSS among three criteria (*p* = 0.873). (**d**) Comparison with DSS among R, BR, and LA in patients without resection: There was no statistical difference of 5-year DSS among three criteria (*p* = 0.053). *DSS:* disease-specific survival, *R*: resectable, *BR*: borderline resectable, *LA*: locally advanced, *MST*: median survival time

As shown in Fig. 4, patients survival was stratified according to the resectability classification: 5-year DSS and MST were 50.3% and not reached in R, 30.0% and 31.4 months in BR, and 16.5% and 22.5 months in LA, respectively. The patients with resection had significantly better prognosis compared to the patients without resection (5-year DSS: 43.8% vs. 5.9%, *p* < 0.001). Interestingly, the patients’ survival did not differ among the three groups when resected.

### Prognostic factors of the patients with resection

**Fig. 5:**
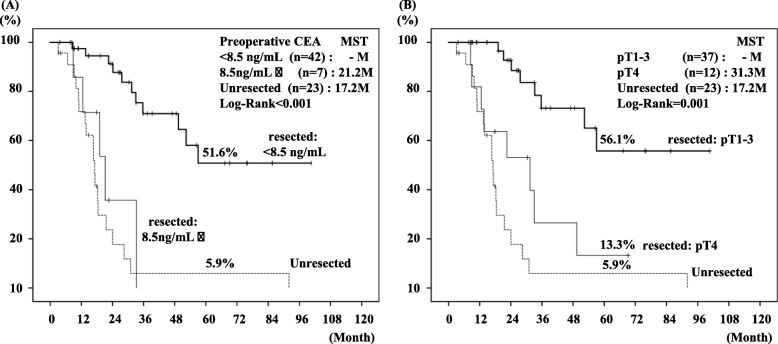
Comparison of DSS curves in resected patients based on the preoperative CEA and pT stage. (**a**) There was significant difference of 5-year DSS between resected patients with preoperative low CEA levels (< 8.5 ng/mL) and high CEA levels (8.5 ng/mL and more) (p < 0.001). Seven patients with preoperative high CEA levels were classified as R (n = 4) and BR (*n* = 3). (**b**) There was significant difference of 5-year DSS between patients with pT1–3 and pT4 (*p* = 0.001). The resected patients with preoperative high CEA levels (more than 8.5 ng/ml) and / or pT4 were no significant differences of DSS compared to unresected patients. *DSS:* disease-specific survival, *R*: resectable, *BR*: borderline resectable, *LA*: locally advanced, *CEA*: carcinoembryonic antigen, *MST*: median survival time

Prognostic factors in the 49 patients with resection were evaluated by uni- and multi-variable analyses **(**Table 5**)**. Univariate analysis identified the following poor prognostic factors: preoperative CEA levels (more than 8.5 ng/ml), G3 histological differentiation, pT4, intrahepatic metastasis, and non R0 resection. Multivariate analysis identified preoperative high CEA levels (more than 8.5 ng/ml) and pT4 as independent poor prognostic factor. The patients with resection showing preoperative high CEA levels (more than 8.5 ng/ml) and pT4 had very poor prognosis, being comparable to the patients without resection **(**Fig. 5**)**.

**Table 5:** Uni- and multi-variable analysis for predictors of disease specific survival of 49 patients with resection

Factors	Univariate analysis				Multivariate analysis	
	No. of patients	5-year survival rate (%)	Median survival time (month)	*p* value	Hazard ratio (95% CI)	*p* value
**Preoperative CA19–9**						
< 25 U/mL	14	59.1	–	0.355		
25 U/mL ≦	35	38.6	49.4			
**Preoperative CEA**						
< 8.5 ng/mL	42	51.6	–	**< 0.001**	**10.516 (2.213–49.971)**	**0.003**
8.5 ng/mL ≦	7	0	21.2			
**Neoadjuvant therapy**						
not done	22	61.1	–	0.561		
done	27	39.2	52.4			
**Adjuvant therapy**						
not done	16	53.0	–	0.897		
done	33	45.7	57.0			
**Combined vascular resection and reconstruction**						
not done	29	31.2	52.4	0.981		
done	20	54.0	–			
**Histological differentiation**						
G1/G2	45	46.7	57.0	**0.001**	7.452 (0.904–61.403)	0.062
G3	3	0	19.1			
**pT factor**						
≦pT3	37	56.1	–	**0.001**	**7.452 (2.348–23.713)**	**0.001**
pT4≦	12	13.3	31.3			
**Lymph node metastasis**						
Negative	26	41.3	57.0	0.389		
Positive	23	52.7	–			
**Intrahepatic metastasis**						
Absent	46	45.4	57.0	**0.033**	0.693 (0.053–9.005)	0.779
Present	3	0	–			
**Curative resection**						
R0	32	61.5	–	**0.037**	2.283 (0.739–7.058)	0.152
R1,2	17	15.1	35.7			

*CEA* carcinoembryonic antigen, *CA19–9* carbohydrate antigen 19–9, *R0* complete resection,*R1* microscopic residual tumor resection, *R2* macroscopic residual tumor resection or distant metastasis (intrahepatic metastasis)

### Outcomes of LA patients

In Table 6, we showed the characteristics of 6 LA patients with resection. The factors of unresectability was vascular factor alone in 2, and biliary factor alone in 4, indicating that no patient had both vascular and biliary factors. Among them, chemotherapy regimen was changed from GS to GC therapy in one patient, and radiotherapy was added in two patients. Operative procedures were right hepatectomy in 3, left hepatectomy in 2, and right trisectionectomy in 1, respectively. Four patients underwent a combined vascular resection and reconstruction: PV alone in 3 and HA in 1. LN metastasis was found in 3 patients (50%), and R0 resection was performed in 4 patients (67%). Among them, three patients died: the one with R0 resection died of liver failure at 8.8 months, the other one with R2 resection died of local tumor growth at 22.5 months, and remained one with R1 resection died of local recurrence at 49.4 months. The other three patients with R0 resection are alive more than 30 months at time of writing.

**Table 6:** Characteristics of 6 locally advanced patients with resection

No	Age (years)	Gender	Factor of unresectability	Adjuvant chemotherapy	Adjuvant radiotherapy	Type of *** Hepatectomy	Vascular resection	pLN	Residual tumor	Survival (status)
1)	50’s	F	Biliary*	G (3 cycles)	36Gy	S1,4,5,6,7,8	PV	1	R2	22.5 m (Dead)
2)	80’s	M	Biliary*	GS (2 cycles)	–	S1,5,6,7,8	–	1	R0	8.8 m (Dead)
3)	60’s	M	Biliary*	GS (2 cycles) + GC (4 cycles)	36Gy	S1,2,3,4	PV	0	R1	49.4 m (Dead)
4)	50’s	M	Biliary*	GS (2 cycles)	–	S1,5,6,7,8	PV	1	R0	47.0 m (Alive)
5)	70’s	F	Vascular (HA)**	GS (10 cycles)	–	S1,5,6,7,8	–	0	R0	49.5 m (Alive)
6)	60’s	M	Vascular (HA)**	GS (4 cycles)	–	S1,2,3,4	HA	0	R0	30.4 m (Alive)

*Biliary factor: biliary margin negative could not be obtained by either side of right or left trisectionectomy

**Vascular factor: Unreconstructible HA and/or PV due to tumor involvement or occlusion

*** Expressed as Couinoud’s hepatic segments resected

*G* gemcitabine, *GS* gemcitabine + TS-1, *GC* gemcitabine + cisplatin, *PV* portal vein, *HA* hepatic artery, *LN* lymph node, *R1* microscopic residual tumor resection, *R2* macroscopic residual tumor resection or distant metastasis (intrahepatic metastasis)

In Table 7, we showed the characteristics of 14 LA patients without resection. The factors of unresectability were vascular factor alone in 7, biliary factor alone in 3, and both vascular and biliary factors in 4, respectively. Among them, chemotherapy could not be introduced due to poor performance status in 2 patients, both of whom died within 11 months. The remaining 14 patients underwent chemotherapy with or without radiotherapy: two are alive at 18.0 and 5.1 months, respectively, the other 9 died within 24 months and the remaining one who underwent chemoradiotherapy had survived for 92 months, dying of local tumor growth.

**Table 7:** Characteristics of 14 locally advanced patients without resection

No	Age (years)	Gender	Factor of unresectability	Chemo-therapy	Radio-therapy	Reason of death	Survival (status)
1)	50’s	M	Vascular (PV and HA)**	GS	50.4Gy	Local tumor growth	92.0 m (Dead)
2)	80’s	M	Biliary* **+** Vascular (PV and HA)**	GS	36Gy	Other disease	14.7 m (Dead)
3)	80’s	F	Biliary* **+** Vascular (HA)**	–	–	Local tumor growth	3.1 m (Dead)
4)	70’s	M	Biliary* **+** Vascular (HA)**	GS	–	Dissemination Liver metastasis	13.9 m (Dead)
5)	70’s	M	Vascular (HA)**	GS	–	Local tumor growth Liver metastasis	21.6 m (Dead)
6)	60’s	M	Biliary* **+** Vascular (HA)**	GS → GC	–	Local tumor growth Lung metastasis	18.5 m (Dead)
7)	60’s	M	Biliary*	GS	–	Local tumor growth Dissemination	18.3 m (Dead)
8)	70’s	M	Biliary*	GS → GC	36Gy	Local tumor growth, Dissemination	16.7 m (Dead)
9)	70’s	M	Vascular (HA)**	–	–	Local tumor growth, Dissemination	10.9 m (Dead)
10)	60’s	F	Biliary*	GS → GC	–	Local tumor growth Liver and bone metastasis	16.7 m (Dead)
11)	40’s	F	Vascular (PV and HA)**	GS → GC	–	Local tumor growth Distant LN metastasis	17.2 m (Dead)
12)	50’s	M	Vascular (PV and HA)**	G → GC	45Gy	Dissemination Lung and bone metastasis	24.0 m (Dead)
13)	70’s	M	Vascular (HA)**	GS → GC	–	–	18.0 m (Alive)
14)	80’s	M	Vascular (HA)**	GS	–	–	5.1 m (Alive)

*Biliary factor: biliary margin negative could not be obtained by either side of right or left trisectionectomy

**Vascular factor: Unreconstructible HA and/or PV due to tumor involvement or occlusion

*G* gemcitabine, *GS* gemcitabine + TS-1, *GC* gemcitabine + cisplatin

LA patients with resection had significantly better prognosis compared to LA patients without resection (MST: 49.4 vs. 18.3 months, *p* = 0.021, Fig. 4).

## Discussion

In the present study, we proposed anatomical resectability classification for patients with localized perihilar cholangiocarcinoma according to surgical points of view from biliary and vascular factors, and the enrolled 72 patients had been classified into the three groups: R (*n* = 29), BR (*n* = 23), and LA (*n* = 20), respectively. Based on this classification and LN status, NAC using 2 cycles GS followed by curative-intent surgery was administered for the 47 patients, and its completion rate was 91.4% (43/47), which was feasible and tolerable. The resection rate was similar between R (89.7%) and BR (73.9%), but much lower in LA (30%). The 5-year DSS was stratified according to the resectability classification: 50.3% in R, 30.0% in BR, and 16.5% in LA, respectively. The patients with resection had significantly better prognosis compared to those without resection (5-year DSS: 43.8% vs. 5.9%). Interestingly, the patients’ survival did not differ among the three groups when resected. Among the 49 patients with resection, preoperative high CEA levels (more than 8.5 ng/ml) and pT4 were identified as independent poor prognosis factors, and the patients with resection showing high CEA levels or pT4 had very poor prognosis, being comparable to the patients without resection.

Patients’ prognosis with perihilar cholangiocarcinoma depends on complete tumor resection, that is, R0 resection. In the absence of widespread disease, the likelihood of achieving R0 resection requires examination of all factors related to local tumor extent. In 1975, Bismuth et al. [20] reported 4 types of biliary stricture based on intraoperative cholangiography [21, 22], which depended on tumor location and extent within the biliary tree. To determine the likelihood of achieving R0 resection, in other words, to predict tumor resectability, additional factors such as vascular involvement and consequent hepatic lobar atrophy should be addressed. In 1998, Blumgart et al. proposed a preoperative staging system to predict unresectability that accounts for local tumor factors including biliary and portal venous involvement, and lobular atrophy [23], and subsequently they reported that this criteria accurately predicted resectability and correlated with survival [24]. Blumgart criteria for unresectablility of perihilar cholangiocarcinoma was based on patients’ factors, local factors, and distant disease. The local factors were defined by the following five factors: 1) hepatic duct involvement up to secondary radicles bilaterally, 2) encasement or occlusion of the main portal vein proximal to its bifurcation, 3) atrophy of one liver lobe with encasement of contralateral portal vein branch, and 4) atrophy of one liver lobe with contralateral secondary biliary radicle involvement. This criteria, however, did not include arterial involvement because of insufficient accuracy of imaging diagnosis at the time of their writing.

With recent advancement in imaging studies such as thin slice dynamic MDCT, we are able to evaluate tumor involvement precisely including artery invasion. In the most recent 8th UICC staging system (2017), arterial involvement is incorporated into T4 stage: tumor invades the main PV or its branches bilaterally or the common hepatic artery; or unilateral second order biliary radicles with contralateral PV or HA involvement. Recently, the development of surgical techniques has enabled us to perform technically demanding procedures including trisectionectomy, and combined PV and/or HA resection. Therefore, using these technically demanding procedures, the tumors determined as unresectable according to Blumgard criteria, Bismuth IV, or UICC T4 stage are not always unresectable, and actually some of them can undergo negative margin resection.

In terms of survey of distant metastasis, preoperative MRI to evaluate intrahepatic metastases or peritoneal dissemination was not performed routinely. Of 54 laparotomy cases in this study (Fig. 3), 4 cases (7.4%) had intrahepatic small metastases and 3 cases (5.5%) had peritoneal dissemination. Several papers reported the usefulness of Gadolinium ethoxybenzyl diethylenetriamine pentaacetic acid-enhanced MR imaging (EOB-MR imaging) compared with MDCT in detecting liver metastasis from colorectal and pancreatic cancers [25, 26]. It was considered that preoperative EOB-MR imaging was useful in detecting small liver metastases.

Regarding the operative procedure for patients of Bismuth type I or II, although limited hilar bile duct resection without major hepatectomy is occasionally underwent, our institution usually employs right hepatectomy with caudate lobectomy, based on previous studies. Ikeyama et al. reported retrospective study using 31 consecutive patients who underwent resection of these types of tumors [27]. R0 resection and survival rates of patients who underwent right hepatectomy with caudate lobectomy (*n* = 18) were significantly better than those of patients who underwent other types of resection (*n* = 13). In that study, most patients did not have invasion of the right hepatic artery, but the distance between the leading edge of the cancer and the outer layer of the hepatic artery was 1 mm in many patients. The authors suggested that the resected margin would have been cancer positive without combined resection of the right hepatic artery. Therefore, they recommend right hepatectomy even when invasion of the right hepatic artery cannot be demonstrated preoperatively by diagnostic imaging. Additionally, 2 small studies also reported that outcomes of limited resection except for those of right hepatectomy with caudate lobectomy were unsatisfactory due to low curative resection rate and survival rate [28, 29]. Further evaluation with larger sample sizes is required to justify right hepatectomy with caudate lobectomy for patients with Bismuth type I or II perihilar cholangiocarcinoma.

In the fields of localized PDAC, the National Comprehensive Cancer Network (NCCN) guidelines for Pancreatic Adenocarcinoma in Version 3. 2019 clearly defines the resectability criteria (R, BR and LA) based on a likelihood of a positive margin resection. The definition of BR-PDAC is a tumor that is at high risk for positive margin resection when surgery is used as an initial treatment. LA-PDAC is defined a tumor not to achieve R0 resection and not to indicate up-front surgery [30]. In the fields of localized perihilar cholangiocarcinoma, however, there is no resectability criteria internationally agreed. Blumgard et al. defined LA (unresectable) perihilar cholangiocarcinoma as mentioned previously. In terms of definition of BR perihilar cholangiocarcinoma, in 2011, Chi et al. firstly reported the anatomical definition of BR perihilar cholangiocarcinoma based on biliary extension alone [31]. They defined that BR perihilar cholangiocarcinoma had a chance of curative resection but an unclear longitudinal tumor extent. In 2017, Matsuyama et al. secondly reported that patients with both regional LN metastasis and vascular invasion were oncologically defined as BR because such patients had dismal outcome [32]. Our new resectability classification is based on PV, HA and biliary involvements according to surgical points of view, and consists of the three categories (R, BR, and LA). This resectability classification is based on the likelihood of an R0 resection.

The role of AC for resected BTC is controversial. Recently, 3 phase-III randomized trials have been explored in the adjuvant setting for BTC [2–4], but a definitive survival advantage has been difficult to prove. However, based on these 3 phase-III randomized trials and 16 retrospective studies, ASCO clinical practice guideline in 2019 presents that patients with resected BTC should be offered adjuvant capecitabine chemotherapy for a duration of 6 months. In contrast, the role of NAC for localized BTC has been explored in several small clinical studies. Jung et al. retrospectively reviewed 57 patients who underwent resection for locally advanced perihilar cholangiocarcinoma, whose definition is compatible to BR or LA according to our resectability classification, and compared the MST between 12 patients with neoadjuvant chemoradiotherapy and 45 without it, showing no significant differences between the two groups (32.9 vs. 27.1 months) [5]. The other retrospective study reviewed 21 patients undergoing down-sizing chemotherapy for locally advanced BTC (ICC in 7, GBC in 6, and ECC in 8), and compared the MST between 8 patients with resection and 13 without resection, showing significant differences between the two groups (19.3 vs. 7.5 months) [7]. However, all of previous studies were retrospective small cohort and lack of a comparable control group, which not allow for a meaningful evaluation of the effects of NAC on prognosis. The reasons for difficulty in establishing valuable effects of NAC are considered that BTC includes cancer of the intrahepatic bile ducts, perihilar and distal cholangiocarcinoma, and the gallbladder, which have different biological characteristics and different effectiveness of chemotherapy. Therefore, a separate study is required for individual type of BTC. Although small number patients with BTC were treated in individual facilities, large-scale clinical studies should be conducted for obtaining the appropriate evidence. In the absence of definitive evidence of NAC for localized BTC, using the large National Cancer Database data, a propensity score matched analysis using resected patients with cholangiocarcinoma indicated that selected 278 patients who received NAC alone had a superior overall survival compared to selected 700 patients who received AC alone (MST: 40.3 vs. 32.8 months) [8]. They implied the benefit of NAC for selected patients with cholangiocarcinoma. However, this study has the problem that about 70% study cohort includes ICC. This is because ICC and ECC in oncologically difference.

It is unclear which patient is most likely to benefit from NAC for perihilar cholangiocarcinoma. Nagino et al. [1] evaluated prognosis factors in the patients who underwent curative-intent surgery for perihilar cholangiocarcinoma and concluded that LN metastasis had the strongest impact on survival among independent prognosis factors such as combined vascular resection and reconstruction. They indicated that the control of LN metastasis was very difficult by surgical treatment alone and that establishment of effective AC was urgently needed. The other study reported that patients with both regional LN metastasis and vascular invasion were candidate for initiating NAC to obtain better outcomes [32]. Therefore, our present study enrolled the patients with clinically obvious LN metastasis, BR, and LA for NAC using GS therapy followed by curative-intent surgery. To the best of our knowledge, our study is the first prospective single arm trial to evaluate NAC for localized perihilar cholangiocarcinoma based on its resectability classification and LN status. As a result, we could demonstrate that curative-intent surgery was an important factor for achieving a good prognosis for perihilar cholangiocarcinoma, even if LA patients. However, multivariate analysis for prognosis factor in the patients with resection identified preoperative high CEA levels (8.5 or more ng/ml) and pT4 as the independent poor prognostic factors. Especially, the patients with resection showing preoperative high CEA levels had very poor prognosis, being comparable to the patients without resection. In these patients, indication of curative-intent surgery should be carefully performed by prolonging preoperative therapy as a watch and wait approach. Additionally, our recent study demonstrated that high tumor budding (TB) can be a novel poor prognostic factor in resected perihilar cholangiocarcinoma regardless of neoadjuvant therapy [33] as well as various type of cancers such as esophageal [34] and pancreas [35]. However, the TB cannot be evaluated before surgery. Therefore, there is an urgent need to identify preoperative predictors of high TB. It is future issue to develop more effective NAC regimen with or without radiotherapy and increase rate of curative-intent surgery in BR and LA patients.

According to the factor analysis for unresectability in 20 LA patients **(**Tables 6 and 7**)**, 6 patients with resection had either one of the vascular or biliary factor, while none of the 4 patients with both vascular and biliary factors could not undergo resection. LA patients with both biliary and vascular factors are not for the candidate of conversion surgery after neoadjuvant GS therapy.

There are several limitations in this study. Study cohort was small and data were collected from a single Japanese center. To evaluate the efficacy of resectability classification and NAC for localized perihilar cholangiocarcinoma patients, prospective randomized multicenter trials are needed.

## Conclusions

NAC based on our resectability classification for patients with localized perihilar cholangiocarcinoma according to surgical points of view from biliary and vascular factors and LN status was feasible and tolerable. NAC enabled the downstaging / downsizing of BR patients and conversion surgery in selected LA patients resulted in improving prognosis.

## Data Availability

The datasets used and/or analyzed during the current study are available from the corresponding author on reasonable request.

## Abbreviations

LN: Lymph node
AC: Adjuvant chemotherapy
BTC: Bile duct cancer
NAC: Neoadjuvant chemotherapy
PDAC: Pancreatic ductal adenocarcinoma
R: Resectable
BR: Borderline resectable
LA: Locally advanced
ERC: Endoscopic retrograde cholangiography
MDCT: Multidetector-row computed tomography
IDUS: Intraductal ultrasonography
PV: Portal vein
HA: Hepatic artery
PET-CT: Positron emission tomography-computed tomography
MR: Magnetic resonance
GS: Gemcitabine plus S-1
GC: Gemcitabine plus cisplatin
CEA: Carcinoembryonic antigen
CA19–9: Carbohydrate antigen 19–9
NLR: Neutrophil-to-lymphocyte ratio
PNI: Prognostic nuritional index
f-rem: Functional remnant liver ratio
KICG: Indocyanine green plasma clearance rate
DSS: Disease-specific survival
MST: Median survival time
TB: Tumor budding
EOB: Gadolinium ethoxybenzyl diethylenetriamine pentaacetic acid-enhanced

## References

[1] Nagino M, Ebata T, Yokoyama Y, Igami T, Sugawara G, Takahashi Y, Nimura Y (2013). Evolution of surgical treatment for perihilar cholangiocarcinoma: a single-center 34-year review of 574 consecutive resections. Ann Surg.

[2] Ebata T, Hirano S, Konishi M, Uesaka K, Tsuchiya Y, Ohtsuka M, Kaneoka Y, Yamamoto M, Ambo Y, Shimizu Y, Ozawa F, Fukutomi A, Ando M, Nimura Y, Nagino M (2018). Bile Duct Cancer Adjuvant Trial (BCAT) study group. Randomized clinical trial of adjuvant gemcitabine chemotherapy versus observation in resected bile ductcancer. Br J Surg.

[3] Edeline J, Benabdelghani M, Bertaut A, Watelet J, Hammel P, Joly JP, Boudjema K, Fartoux L, Bouhier-Leporrier K, Jouve JL, Faroux R, Guerin-Meyer V, Kurtz JE, Assénat E, Seitz JF, Baumgaertner I, Tougeron D, de la Fouchardière C, Lombard-Bohas C, Boucher E, Stanbury T, Louvet C, Malka D, Phelip JM (2019). Gemcitabine and oxaliplatin chemotherapy or surveillance in resected biliary tract cancer (PRODIGE 12-ACCORD 18-UNICANCER GI): a randomized phase iii study. J Clin Oncol.

[4] Primrose JN, Fox RP, Palmer DH, Malik HZ, Prasad R, Mirza D, Anthony A, Corrie P, Falk S, Finch-Jones M, Wasan H, Ross P, Wall L, Wadsley J, Evans JTR, Stocken D, Praseedom R, Ma YT, Davidson B, Neoptolemos JP, Iveson T, Raftery J, Zhu S, Cunningham D, Garden OJ, Stubbs C, Valle JW, Bridgewater J (2019). BILCAP study group. Capecitabine compared with observation in resected biliary tract cancer (BILCAP): a randomised, controlled, multicentre, phase 3 study. Lancet Oncol.

[5] Jung JH, Lee HJ, Lee HS, Jo JH, Cho IR, Chung MJ, Park JY, Park SW, Song SY, Bang S (2017). Benefit of neoadjuvant concurrent chemoradiotherapy for locally advanced perihilar cholangiocarcinoma. World J Gastroenterol.

[6] Le Roy B, Gelli M, Pittau G, Allard MA, Pereira B, Serji B, Vibert E, Castaing D, Adam R, Cherqui D, Sa CA (2018). Neoadjuvant chemotherapy for initially unresectable intrahepatic cholangiocarcinoma. Br J Surg.

[7] Kato A, Shimizu H, Ohtsuka M, Yoshidome H, Yoshitomi H, Furukawa K, Takeuchi D, Takayashiki T, Kimura F, Miyazaki M (2013). Surgical resection after downsizing chemotherapy for initially unresectable locally advanced biliary tract cancer: a retrospective single-center study. Ann Surg Oncol.

[8] Yadav S, Xie H, Bin-Riaz I, Sharma P, Durani U, Goyal G, Borah B, Borad MJ, Smoot RL, Roberts LR, Go RS, McWilliams RR, Mahipal A (2019). Neoadjuvant vs. adjuvant chemotherapy for cholangiocarcinoma: a propensity score matched analysis. Eur J Surg Oncol.

[9] Kobayashi M, Mizuno S, Murata Y, Kishiwada M, Usui M, Sakurai H, Tabata M, Ii N, Yamakado K, Inoue H, Shiraishi T, Yamada T, Isaji S (2014). Gemcitabine-based chemoradiotherapy followed by surgery for borderline resectable and locally unresectable pancreatic ductal adenocarcinoma: significance of the CA19-9 reduction rate and intratumoral human equilibrative nucleoside transporter 1 expression. Pancreas..

[10] Wolpin BM, Mayer RJ (2010). A step forward in the treatment of advanced biliary tract cancer. N Engl J Med.

[11] Aloia TA, Charnsangavej C, Faria S, Ribero D, Abdalla EK, Vauthey JN, Curley SA (2007). High-resolution computed tomography accurately predicts resectability in hilar cholangiocarcinoma. Am J Surg.

[12] Japan Pancreas Society (2017). Classification of pancreatic carcinoma, 4th English Edition, Kanehara, Tokyo.

[13] Morizane C, Okusaka T, Mizusawa J, Takashima A, Ueno M, Ikeda M, Hamamoto Y, Ishii H, Boku N, Furuse J (2013). Randomized phase II study of gemcitabine plus S-1 versus S-1 in advanced biliary tract cancer: a Japan Clinical Oncology Group trial (JCOG 0805). Cancer Sci.

[14] Sasaki T, Isayama H, Nakai Y, Ito Y, Yasuda I, Toda N, Kogure H, Hanada K, Maguchi H, Sasahira N, Kamada H, Mukai T, Okabe Y, Hasebe O, Maetani I, Koike K (2013). A randomized phase II study of gemcitabine and S-1 combination therapy versus gemcitabine monotherapy for advanced biliary tract cancer. Cancer Chemother Pharmacol.

[15] Ichikawa K, Mizuno S, Hayasaki A, Kishiwada M, Fujii T, Iizawa Y, Kato H, Tanemura A, Murata Y, Azumi Y, Kuriyama N, Usui M, Sakurai H, Isaji S (2019). Prognostic nutritional index after chemoradiotherapy was the strongest prognostic predictor among biological and conditional factors in localized pancreatic ductal adenocarcinoma patients. Cancers.

[16] Sumiyoshi T, Shima Y, Okabayashi T, Kozuki A, Hata Y, Noda Y, Kouno M, Miyagawa K, Tokorodani R, Saisaka Y, Tokumaru T, Nakamura T, Morita S (2016). Liver function assessment using 99mTc-GSA single-photon emission computed tomography (SPECT)/CT fusion imaging in hilar bile duct cancer: A retrospective study. Surgery..

[17] Yokoyama Y, Nishio H, Ebata T, Igami T, Sugawara G, Nagino M (2010). Value of indocyanine green clearance of the future liver remnant in predicting outcome after resection for biliary cancer. Br J Surg.

[18] Miyazaki M, Kimura F, Shimizu H, Yoshidome H, Otsuka M, Kato A, Hideyuki Y, Nozawa S, Furukawa K, Mituhashi N, Takeuchi D, Suda K, Takano S (2008). Extensive hilar bile duct resection using a transhepatic approach for patients with hepatic hilar bile duct diseases. Am J Surg.

[19] Clavien PA, Barkun J, de Oliveira ML, Vauthey JN, Dindo D, Schulick RD, de Santibañes E, Pekolj J, Slankamenac K, Bassi C, Graf R, Vonlanthen R, Padbury R, Cameron JL, Makuuchi M (2009). The Clavien-Dindo classification of surgical complications: five-year experience. Ann Surg.

[20] Bismuth H, Corlette MB (1975). Intrahepatic cholangioenteric anastomosis in carcinoma of the hilus of the liver. Surg Gynecol Obstet.

[21] Bismuth H, Castaing D, Traynor O (1988). Resection or palliation: priority of surgery in the treatment of hilar cancer. World J Surg.

[22] Burke EC, Jarnagin WR, Hochwald SN, Pisters PW, Fong Y, Blumgart LH (1998). Hilar Cholangiocarcinoma: patterns of spread, the importance of hepatic resection for curative operation, and a presurgical clinical staging system. Ann Surg.

[23] Jarnagin WR, Fong Y, DeMatteo RP, Gonen M, Burke EC, Bodniewicz BSJ, Youssef BAM, Klimstra D, Blumgart LH (2001). Staging, resectability, and outcome in 225 patients with hilar cholangiocarcinoma. Ann Surg.

[24] Tempero MA, Malafa MP, Chiorean EG, Czito B, Scaife C, Narang AK, Fountzilas C, Wolpin BM, Al-Hawary M, Asbun H, Behrman SW, Benson AB, Binder E, Cardin DB, Cha C, Chung V, Dillhoff M, Dotan E, Ferrone CR, Fisher G, Hardacre J, Hawkins WG, Ko AH, LoConte N, Lowy AM, Moravek C, Nakakura EK, O’Reilly EM, Obando J, Reddy S, Thayer S, Wolff RA, Burns JL, Zuccarino-Catania G (2019). PancreaticAdenocarcinoma, Version 1. J Natl Compr Cancer Netw.

[25] Ito T, Sugiura T, Okamura Y, Yamamoto Y, Ashida R, Aramaki T, Endo M, Uesaka K (2017). The diagnostic advantage of EOB-MR imaging over CT in the detection of liver metastasis in patients with potentially resectable pancreatic cancer. Pancreatology..

[26] Seo N, Park MS, Han K, Lee KH, Park SH, Choi GH, Choi JY, Chung YE, Kim MJ, Kim HJ, Lee SS, Byun JH, Kim JC, Yu CS, Park SH, Kim AY, Ha HK (2015). Incremental value of liver MR imaging in patients with potentially curable colorectal hepatic metastasis detected at CT: a prospective comparison of diffusion-weighted imaging, gadoxetic acid-enhanced MR imaging, and a combination of both MR techniques. Radiology..

[27] Ikeyama T, Nagino M, Oda K, Ebata T, Nishio H, Nimura Y (2007). Surgical approach to bismuth Type I and II hilar cholangiocarcinomas: audit of 54 consecutive cases. Ann Surg.

[28] Neuhaus P, Jonas S, Bechstein WO (1999). Extended resections for hilar cholangiocarcinoma. Ann Surg.

[29] Seyama Y, Kubota K, Sano K (2003). Long-term outcome of extended hemihepatectomy for hilar bile duct cancer with no mortality and high survival rate. Ann Surg.

[30] Isaji S, Mizuno S, Windsor JA, Bassi C, Fernández-Del Castillo C, Hackert T, Hayasaki A, Katz MHG, Kim SW, Kishiwada M, Kitagawa H, Michalski CW, Wolfgang CL (2018). International consensus on definition and criteria of borderline resectable pancreatic ductal adenocarcinoma 2017. Pancreatology..

[31] Choi ER, Chung YH, Lee JK, Lee KT, Lee KH, Choi DW, Choi SH, Heo JS, Jang KT, Park SM, Lim JH (2011). Preoperative evaluation of the longitudinal extent of borderline resectable hilar cholangiocarcinoma by intraductal ultrasonography. J Gastroenterol Hepatol.

[32] Matsuyama R, Morioka D, Mori R, Yabushita Y, Hiratani S, Ota Y, Kumamoto T, Endo I (2019). Our rationale of initiating neoadjuvant chemotherapy for hilar cholangiocarcinoma: a proposal of criteria for “borderline resectable” in the field of surgery for hilar cholangiocarcinoma. World J Surg.

[33] Ito T, Kuriyama N, Kozuka Y, Komatsubara H, Ichikawa K, Noguchi D, Hayasaki A, Fujii T, Iizawa Y, Kato H, Tanemura A, Murata Y, Kishiwada M, Mizuno S, Usui M, Sakurai H, Isaji S (2020). High tumor budding is a strong predictor of poor prognosis in the resected perihilar cholangiocarcinoma patients regardless of neoadjuvant therapy, showing survival similar to those without resection. BMC Cancer.

[34] Miyata H, Yoshioka A, Yamasaki M (2009). Tumor budding in tumor invasive front predicts prognosis and survival of patients with esophageal squamous cell carcinomas receiving neoadjuvant chemotherapy. Cancer..

[35] Karamitopoulou E, Zlobec I, Born D (2013). Tumour budding is a strong and independent prognostic factor in pancreatic cancer. Eur J Cancer.

